# Long-term results of minimally invasive stand-alone bi-atrial surgical ablation with a bipolar ablation device for persistent and longstanding persistent AF: a single-center case series of 91 patients

**DOI:** 10.1186/s13019-016-0416-0

**Published:** 2016-02-02

**Authors:** Vilius Janusauskas, Lina Puodziukaite, Vyte Valerija Maneikiene, Gitana Zuoziene, Greta Radauskaite, Greta Burneikaite, Robertas Stasys Samalavicius, Sigita Aidietiene, Kestutis Rucinskas, Audrius Aidietis

**Affiliations:** Department of Cardiovascular Medicine, Vilnius University, Universiteto street 3, Vilnius, Lithuania; Centre of Anesthesia, Intensive Care, and Pain Management, Department of Intensive Care, Vilnius University, Universiteto street 3, Vilnius, Lithuania

**Keywords:** Minimally invasive, Surgical ablation, Lone atrial fibrillation

## Abstract

**Background:**

Minimally invasive surgical treatment of lone atrial fibrillation (AF) is an alternative for AF that is refractory to medical treatment. We present long-term results of standalone surgical ablation of AF using a bipolar ablation device in 91 consecutive patients.

**Methods:**

This was an observational, retrospective study of 91 patients (77 % males; mean age, 53 ± 10 years [range, 23–75 years]) who underwent minimally invasive standalone surgical ablation of persistent and longstanding persistent AF using a bipolar ablation device from 2008 to 2014. Mean follow-up was 60 ± 21 months. The absence of arrhythmia was confirmed at 3, 6, and 12 months, and annually thereafter, with 24-hour Holter monitoring.

**Results:**

The mean duration of preoperative AF was 6.5 ± 5.4 years. Persistent AF was present in 86 % of patients and longstanding persistent AF in 14 %. Mean left atrial diameter was 4.3 ± 0.8 cm. There were two postoperative strokes (2 %) and three conversions to median sternotomy (3 %). Permanent pacemakers were implanted in six (7 %) patients. There were no intra- or postoperative deaths. At 1, 2, 3, 4, and 5 years postoperatively, freedom from AF was 59, 45, 41, 38, and 38 % of patients, respectively. The failure to achieve pulmonary vein isolation was the only independent predictor of long-term recurrence of AF (HR −3 [95 % CI 1,858 to 8,586], *p* = 0,001). There was a tendency towards higher rates of SR at long term follow up in patients with pulmonary vein isolation if division of ligament of Marshall was performed (HR - 2 [95 % CI 0.987 to 4,202], *p* = 0,067).

**Conclusions:**

In the present series, the efficacy of epicardial surgical ablation was similar to that reported previously. The rate of arrhythmia recurrence increased over time. Achieving pulmonary vein isolation is essential to AF elimination. The division of ligament of Marshall could contribute to improved rates of SR restoration in patients with persistent or long-standing persistent AF if PVI is achieved.

## Background

Surgery is a recognized treatment option for patients with atrial fibrillation (AF) [1–3]. Recent guidelines representing collective experience have become more liberal in patient selection for surgical treatment of lone AF [2]. The 2012 HRS/ EHRA/ECAS Expert Consensus Statement on Catheter and Surgical Ablation of Atrial Fibrillation [2] allows consideration of surgical intervention to treat AF if a patient prefers surgical ablation, even without failed catheter ablation. Thus, surgical treatment for lone AF has increased [4].

Some centers report excellent long-term results, with freedom from AF in up to 85 % of patients [4–6]. However, in a systematic review of minimally invasive surgical treatment for AF by Je et al., most of the included studies presented data for a mean follow-up of about 2 years [7]. We report the single-center, long-term results of bi-atrial minimally invasive surgical ablation of lone AF with a bipolar radiofrequency ablation device.

## Methods

This was an observational, retrospective study. From 2008 to 2014, 91 patients underwent video-assisted standalone bipolar radiofrequency ablation for non-valvular persistent or longstanding persistent AF at our institution. Indications for procedures were: AF burden (European Heart Rhythm Association [2] class III–IV), ineffective medical treatment, and patient’s preference to undergo surgical intervention [2]. Per recent guidelines, we defined AF as persistent, or longstanding persistent [1]. The same surgeon performed all procedures. The surgical technique was the same for all patients and included a persistent attempt to achieve pulmonary vein isolation (PVI) after the importance of PVI was noted in initial patient group. A bipolar radiofrequency clamp (Cardioblate® Gemini® Surgical Ablation System; Medtronic, Inc., Minneapolis, MN, USA) was used in all cases.

### Surgical technique

#### Patient positioning and anesthesia

The patient was positioned supine with the arms at the sides. A foam roll approximately 5 cm in diameter was placed under the shoulders to improve exposure. General anesthesia with a double-lumen endotracheal tube was used for the procedure. Routine cardiac monitoring was conducted throughout the procedure, including electrocardiography and invasive radial arterial and central venous pressures. A Foley catheter was placed to monitor diuresis. External defibrillation pads were routinely placed. Transesophageal echocardiography (TEE) was used to rule out thrombus of the atrial appendage and to evaluate the quality of its closure.

#### Right-side access

Single-lung ventilation of the left lung was established. A 4-cm thoracotomy incision was performed at the 4th intercostal space between the anterior and midaxillary lines. The layers were dissected and the parietal pleura opened. A pericardiotomy was performed 1–2 cm anterior to the phrenic nerve. The heart was pulled closer to the incision with retraction sutures, which were left on clips. The oblique pericardial sinus was entered using blunt dissection between the inferior pulmonary vein and the inferior vena cava. A gastric probe was pushed between the inferior pulmonary vein and the inferior vena cava. The pericardial reflection between the superior pulmonary vein and superior vena cava was dissected. A Navigator® Tissue Dissector (Medtronic, Inc.) was introduced and pushed between the superior right pulmonary vein and the superior vena cava to enter the transverse sinus. A guidewire was pushed into the transverse sinus, and the tissue dissector was removed, leaving the guidewire in place. Tension on the pericardial retraction sutures was released.

#### Left-side access

A 5-cm thoracotomy incision was performed in the 3rd intercostal space between the anterior and midaxillary lines. Single-lung ventilation of the right lung was established. The layers were dissected and the parietal pleura opened. A pericardiotomy was performed 1–2 cm posterior to the phrenic nerve. The heart was pulled closer to the incision with retraction sutures, which were left on clips. The guidewire and gastric tube, which had been pushed in from the right side, were pulled, leaving them on the posterior surface of the heart, above and below the pulmonary veins.

The plastic guides included in the Cardioblate® Gemini® Surgical Ablation System set (Medtronic, Inc.) were pulled out using the gastric tube and guidewire. One plastic guide was left above the pulmonary veins and another below the pulmonary veins. The bipolar radiofrequency ablation clamp from the Cardioblate® Gemini® Surgical Ablation System was positioned. After a series of three ablations, the direction of the bipolar ablation device was changed and a second series of three ablations was performed from another side of the chest. After the procedure, exit block of the PVI box created by the ablation lines was checked in all cases. Each pulmonary vein above the ablation line was paced with a rate higher than the patient’s heart rate, and 20-mA stimuli were used to confirm exit block. The standard ablation with three applications from left and right heart sides did not succeed in achieving PVI in 35 (38 %) patients. If PVI was not achieved, individual ablation of each of the four pulmonary veins was performed. This was performed in 24 (26 %) of our patients.

#### Right atrial ablation

**Fig. 1:**
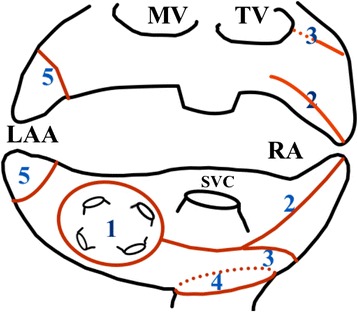
Ablation lines. Pulmonary vein isolation (1); right atrial ablation: line from the RAA targeting the intraatrial septum between the right pulmonary veins (2); line from the lateral part of the right atrium toward the TV annulus (3); circular line at the ostium of the inferior vena cava (4); ligation of the left atrial appendage (5). *LAA* left atrial appendage, *MV* mitral valve, *PV* pulmonary veins, *RAA* right atrial appendage, *SVC* superior vena cava, *TV* tricuspid valve

After PVI was achieved, right atrial ablation was performed. The ablation lines are showed in Fig. 1. Using the bipolar ablation device, additional lines were created on the right atrium: a longitudinal line from the right atrium appendage targeting the intraatrial septum between the right pulmonary veins; a line from the lateral part of the right atrium toward the tricuspid valve annulus; and a circular line at the ostium of the inferior vena cava. These ablation lines were targeting cavotricuspid isthmus. Although there are numerous evidences showing triggers coming from superior vena cava, superior vena cava was not ablated to avoid damage to sinus node. Linear block after ablation has not been checked.

#### Additional procedures

Ligation of the left atrial appendage and division of ligament of Marshall. Ligation of left atrial appendage was performed with 2/0 polyester suture using knot pusher. Two ligatures were placed: one at the basis of the atrial appendage, another – about 7 mm distally from the first ligature. End of atrial appendage was cut to evacuate residual blood from appendage and check ligation quality.

### Postoperative care and follow-up

Clinical demographic and postoperative outcome variables were collected retrospectively and prospectively. Anticoagulation with warfarin was initiated in all patients in the early postoperative period. Class I (propafenone), class II (beta blockers), or class III (amiodarone, sotalol, dronedarone) antiarrhythmic drugs (AADs) were initiated if arrhythmias were noted during the postoperative course. Patients with postoperative atrial tachyarrhythmias were electrically cardioverted if medical conversion was not effective. If patients were in SR and free of atrial tachyarrhythmias on prolonged monitoring (≥6 months) after the procedure, AADs were discontinued. If CHA2DS2-VASc score was less than 2, warfarin was discontinued. TEE was performed before discontinuation of warfarin to rule out atrial thrombus. Follow-up was conducted at 3, 6, and 12 months and annually thereafter. At each visit, history, physical examination, electrocardiogram, and 24-hour Holter monitoring or pacemaker interrogation were obtained. 24-hour Holter monitoring was not performed on 3-month follow up and if patient had arrhythmia on ECG. There were 222 Holter monitoring’s performed in reported patient group, and 27 (12 %) of them have revealed silent arrhythmia. Recurrence was defined as any episode of AF, atrial flutter, or atrial tachycardia that lasted longer than 30 s [8, 9]. Episodes of AF or atrial flutter were treated with AADs, electrical cardioversion, or catheter ablation using a CARTO® Thermocool Catheter (Biosense Webster, Inc.; Diamond Bar, CA, USA). Treatment was considered successful only if the patient was free of AF and off class I or class III AADs.

### Statistical analysis

Categorical variables are presented as proportions and were compared with Pearson’s χ2 test and Fisher’s exact test. Normally distributed continuous variables are expressed as means ± SD and were compared with Student’s unpaired t-test. Non-normally distributed continuous variables were expressed median and range and were compared with Mann–Whitney U-test. Kaplan-Meier method was used to estimate arrhythmia and AAD free survival. Log rank test was used to compare Kaplan-Meier curves. Univariate and multivariate predictors of recurrent AF were evaluated using Cox proportional hazards regression model. Variables were included in the multivariable analysis using a forward stepwise procedure with criteria of *P* < 0,05 for inclusion and *P* > 0,10 for removal from the model. All reported *P* values were two-sided. A value of *P* < 0,05 was considered to indicate statistical significance. A value of 0,05 < *P* < 0,1 was considered to indicate tendency. IBM SPSS for Macintosh software, Version 20.0 (IBM Corp., Armonk, NY, USA) was used for statistical analyses.

## Results

Patient characteristics, including sex and mean age, as well as preoperative clinical data, including distribution of type and mean duration of AF, atrial dimensions, ablation history, and comorbidities, are presented in Table 1. The average patient age was 53 years, range 23–75 years. Twenty-one percent of patients had failed a previous catheter ablation, and 17 (19 %) had thyroid dysfunction due to amiodarone use. Patients with long standing persistent AF had longer AF duration and larger left atria. There were no other significant differences between those patient groups (Table 1).

**Table 1:** Patient characteristic

	Overall (*N* = 91)	Persistent AF (*N* = 78)	Long standing persistent AF (*N* = 13)	*P* value
Follow up (months)	60 ± 21	60 ± 21	63 ± 19	0.569
Age (years)	53 ± 10	53 ± 9	52 ± 11	0.661
Patients < 65 years old	80 (87 %)	69 (89 %)	11 (85 %)	0.694
Patients 65–74 years old	10 (11 %)	8 (10 %)	2 (15 %)	0.631
Patients > 65–74 years old	1 (1 %)	1 (1 %)	0 (0 %)	1.000
Female gender	21 (23 %)	19 (24 %)	2 (15 %)	0.726
LV dysfunction	11 (12 %)	8 (10 %)	3 (23 %)	0.189
Hypertension	73 (80 %)	62 (80 %)	11 (85 %)	1.000
Diabetes Mellitus	5 (6 %)	4 (5 %)	1 (8 %)	0.546
History of TIA	5 (5 %)	4 (8 %)	1 (8 %)	1.000
Stroke on adequate anticoagulation	2 (2 %)	2 (2 %)	0 (0 %)	1.000
Peripheral vascular disease	4 (4 %)	4 (5 %)	0 (0 %)	1.000
CHA2DS2-VASc Score = 0	9 (10 %)	8 (10 %)	1 (8 %)	0.774
CHA2DS2-VASc Score = 1	47 (52 %)	40 (51 %)	7 (54 %)	0.864
CHA2DS2-VASc Score ≥2	35 (38 %)	30 (38 %)	4 (30 %)	0.596
AF duration (months)	73 ± 66	40 ± 37	79 ± 69	0.006
Failed catheter ablation	10 (21 %)	17 (22 %)	2 (15 %)	0.598
Failed catheter ablation >1 time	9 (10 %)	7 (10 %)	2 (15 %)	0.474
Median LVEF (%)	55 (30–65)	55 (30–65)	55 (40–55)	0.293
LA diameter (cm)	4.3 ± 0.5	4.2 ± 0.5	4.7 ± 0.4	0.046
Operative				
Median operation duration (min)	180 (90–280)	180 (90–280)	170 (120–240)	0.497
Ablation time (min)	14 ± 4	13 ± 4	15 ± 6	0.772
PV Ablation time (min)	10 ± 3	10 ± 3	10 ± 5	0.864
RA Ablation time (min)	5 ± 3	6 ± 4	4 ± 2	0.293
Division of ligament of Marshall	61 (67 %)	55 (71 %)	6 (46 %)	0.112
Box lesion around PVs (exit block achieved)	80 (88 %)	70 (90 %)	10 (77 %)	0.189

Data are presented as mean ± standard deviation, median and range or percent

*AF* atrial fibrillation, *LA* left atrial, *LV* left ventricle, *PV* pulmonary vein, *PVI* PV isolation, *RA* right atrial, *TIA* transient ischemic attack

Median operative duration was 180 (range, 90–280 min). PVI was achieved in 88 % of patients and proven by checking for exit block. In 24 (26 %) of patients, PVI of each pulmonary vein was performed. Table 1 presents intraoperative details.

In one patient it was impossible to perform PVI with the available equipment, as there was ST-segment depression and a significant drop in blood pressure as soon as the pulmonary veins were clamped. Postoperative contrast-enhanced computed tomography suggested that the left main stem might have become compressed during application of the bipolar ablation-device clamps.

Left atrial appendage was not excluded in one patient. The patient had fragile tissues, LA appendage was short with wide base, and it was surgeon’s decision to abandon atrial appendage ligation to avoid risk of bleeding.

Median stay in the intensive care unit was 1 day (range, 1–7 days), and median length of hospitalization was 12 days (range, 2–32 days). There were two postoperative strokes (2 %) and three conversions to median sternotomy (3 %): one because of damage to the pulmonary artery, one because of damage to the left atrial appendage, and one in the intensive care unit because of tamponade caused by bleeding from the surface of the right ventricle. One wound revision was required because of bleeding into soft tissues. One patient (1 %) developed a superficial wound infection. Three patients (3 %) required early postoperative permanent pacemaker implantation for chronotropic incompetence. Early postoperative atrial arrhythmias were documented in 35 % of patients, most frequently first week postoperatively. There were no intra- or postoperative deaths.

### Late follow-up

**Fig. 2:**
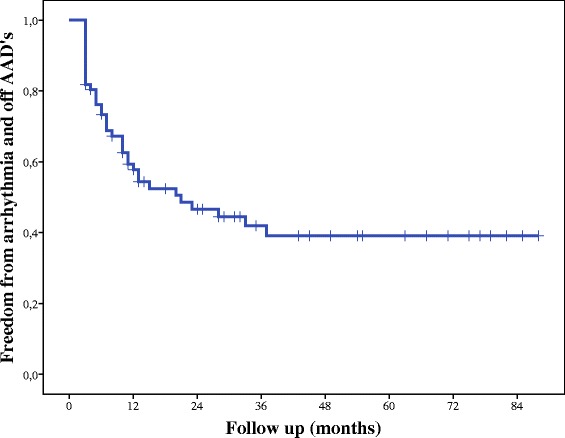
Freedom from arrhythmia off AADs at follow-up. *AAD* antiarrhythmic drug

Mean follow-up was 5 years (range, 1–7 years), and 42 out of 49 patients (87 %) completed 5 years of follow-up. At 3, 6, 12, 24, 36, 48 and 60 months, freedom from AF was seen in 82, 75, 59, 45, 41, 38, and 38 % of patients, respectively. Five years postoperatively, 38 % of patients were arrhythmia- and AAD-free (Fig. 2).

Nine patients (9 %) underwent a CARTO® ablation procedure postoperatively. Five patients (6 %) in whom PVI was achieved underwent CARTO® ablation, compared with 5 (45 %) of those in whom PVI was not achieved (RR - 7 [95 % CI 2,5 to 21,1], *p* = 0.001). Three patients (3 %) required permanent pacemaker implantation at late follow up. Two patients have been diagnosed with sick sinus syndrome 30 and 36 months after the procedure; one patient underwent postoperative permanent pacemaker implantation and atrioventricular-node ablation for uncontrolled, symptomatic AF 26 months after the procedure.

All patients with AAD-free sinus or atrially-paced rhythm were off warfarin after six months after the procedure if CHA_2_DS_2_-VASc score was less than 2. Eight patients out of sixteen (50 %) who were with SR were off warfarin at five years follow up. Recurrent arrhythmias were AF (75 %) and atrial flutter (25 %). Late postoperative complications included pulmonary vein stenosis in one patient (1 %) and bilateral lung hernia in one patient (1 %). Pulmonary vein stenosis did not require any intervention. There were no deaths at late follow-up.

### Predictors of arrhythmia recurrence

**Fig. 3:**
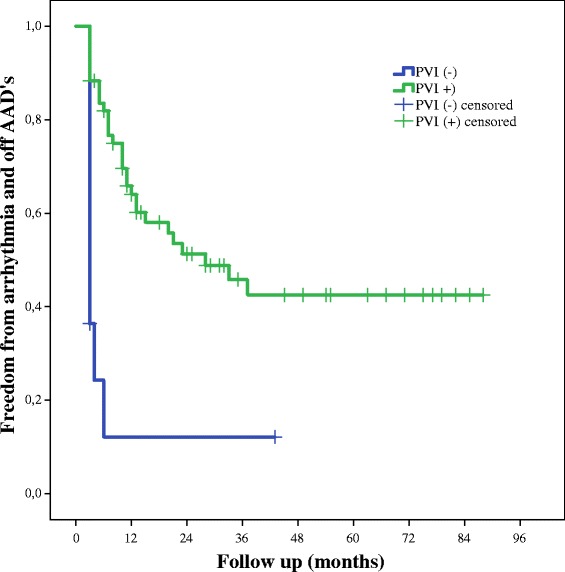
Freedom from arrhythmia off AADs at follow-up in patients with and without achievement of PVI. *AAD* antiarrhythmic drug, *PVI(+)* pulmonary vein isolation achieved, *PVI(−)* pulmonary vein isolation not achieved

In our patient cohort any of preoperative factors (age, gender, AF duration, long term persistent AF, hypertension, vascular disease, diabetes mellitus, thyroid dysfunction, presence of heart failure, LA size) could not predict reoccurrence of AF in Cox regression analysis (*p* values above 0,05). Only pulmonary vein isolation was related with SR at follow up (HR −3 [95 % CI 1,858 to 8,586], *p* = 0,001) in multivariate Cox regression analysis. At last follow up 47 (59 %) patients with PVI were in sinus rhythm and only 1 (9 %) patient without PVI was in SR. The log rank test was used to compare Kaplan – Meier arrhythmia and AAD free survival curves and it confirmed that AF recurrence was significantly less common in patients with achieved PVI (χ2 = 16, *p* < 0,005) (Fig. 3).

To determine predictors of success in restoring SR only patients with achieved PVI were analyzed (80 patients). Univariate predictors of long-term recurrent AF were absence of division of ligament of Marshall (HR −2,3 [95 % CI 1,035 to 5,202], *p* = 0,041), age (HR −1,058 [95 % CI 1,008 to 1,110], *p* = 0,023) and presence of thyroid dysfunction (HR −2,538 [95 % CI 1,090 to 6,191], *p* = 0,030). Multivariate Cox regression analysis revealed that there was a tendency towards higher rates of SR at long term follow up in patients with division of ligament of Marshall (HR - 2 [95 % CI 0.987 to 4,202], *p* = 0,067). Thirty five patients (59 %) were in SR at last follow if PVI was achieved and ligament of Marshall was divided up and 10 (48 %) patients were in SR if ligament of Marshall was not divided.

**Fig. 4:**
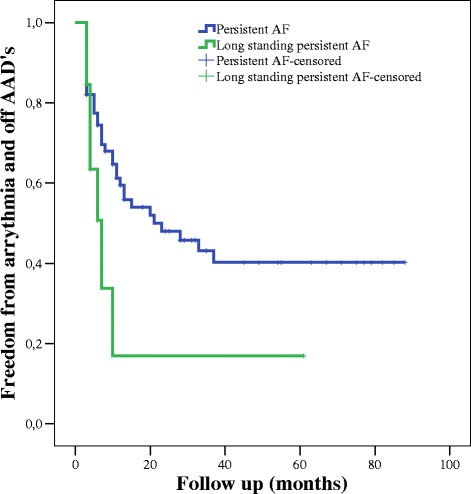
Freedom from AF and off AADs at follow-up in patients with persistent and longstanding persistent AF. *AAD* antiarrhythmic drug, *AF* atrial fibrillation

The persistent AF outcome seems better than long term persistent AF, but not significant statistically (χ2 = 1,075, *p* < 0,3), which might be secondary to small patient number (Fig. 4).

## Discussion

This study has some limitations. This is a single-center, observational, retrospective study, and 13 % of patients were lost to follow-up at 5 years. Implantable loop monitoring, which is more precise in diagnosing arrhythmias and may have yielded different results [10], was not used in our study.

Figure 2 shows that the effectiveness of the procedure diminished with time; 12 months postoperatively, 59 % of patients were arrhythmia- and AAD-free, but that number declined to just 38 % at the 5-year follow-up (Fig. 2). Our early results are comparable to those of other centers [7, 11], where freedom from arrhythmias and AADs was achieved in 72 % of patients at 12 months in cases in which epicardial surgical ablation was used. Long-term results have been reported only rarely, but demonstrate a 5-year AF-free survival of 28 % for persistent, and 29 % for longstanding AF if only PVI and ganglionic plexus ablation was used [11]. Some studies of catheter ablation report 20–45 % success with single or multiple ablations for longstanding persistent AF at the 5-year follow-up [12].

Epicardial surgical ablation is used in very different ways and may vary from PVI only, to PVI with an additional ablation line to the left atrial appendage and/or ganglion ablation and/or right atrial ablation [7, 9, 13]. Most of the radiofrequency surgical ablations on beating heart are confined to left atrium [14, 15]. To date, however, whether a right atrium ablation is necessary remains controversial. There are numerous studies confirming that biatrial ablation is more effective in controlling persistent and longstanding persistent AF than procedures confined to left atrium only [14–18]. Biatrial ablation strategy was chosen in our institution. The right atrium lesion set (Fig. 1) could be safely carried out using available bipolar radiofrequency device. Right atrial ablation may decrease the amount of tissue hosting a variety of triggers for AF and eliminate the substrate of atrial tachyarrhythmia’s, which may improve the clinical outcome in patients with non-paroxysmal AF [16, 19]. In general, however, epicardial ablation lacks a lesion across the mitral valve isthmus [7] and the effect of our ablation lines targeting the tricuspid isthmus is debatable. The endocardial Cox maze procedure enables the creation of those lines and is more effective than epicardial ablation [7]. The hybrid surgical ablation as well enables the creation of those lines but overall results seems to be similar to epicardial surgical ablation [7].

The importance of ligament of Marshall to development of AF is well described [20]. Ligament of Marshall can be source of ectopic activity or reentry substrate AF [21–23]. The division of ligament of Marshall is save and feasible procedure though the left side chest access and in our study was tendency towards higher rates of SR after surgical ablation of persistent or long standing persistent AF if PVI was achieved.

## Conclusion

In conclusion, the lesion set used in our institution provides efficacy similar to that reported previously. It is crucial to check and achieve PVI during surgical epicardial ablation for atrial fibrilation. In our patient series standard ablation with three applications from both heart sides did not succeed in achieving PVI in 35 (38 %) patients. Failing to achieve PVI leads to very low rates of SR restoration. Achieving PVI should be pursued aggressively, and the procedure continued, until PVI is achieved or patient safety is endangered. The division of ligament of Marshall could contribute to improved rates of SR restoration in patients with persistent or long-standing persistent AF if PVI is achieved.

### Consent

Written informed consent was obtained from the patients for publication of this case report and any accompanying images. A copy of the written consent is available for review by the Editor-in-Chief of this journal.
